# S1-bZIP Transcription Factors Play Important Roles in the Regulation of Fruit Quality and Stress Response

**DOI:** 10.3389/fpls.2021.802802

**Published:** 2022-01-14

**Authors:** Hong Wang, Yunting Zhang, Ayla Norris, Cai-Zhong Jiang

**Affiliations:** ^1^Jiangsu Key Laboratory for Horticultural Crop Genetic Improvement, Institute of Pomology, Jiangsu Academy of Agricultural Sciences, Nanjing, China; ^2^Department of Plant Sciences, University of California at Davis, Davis, CA, United States; ^3^College of Horticulture, Sichuan Agricultural University, Chengdu, China; ^4^Crops Pathology and Genetics Research Unit, United States Department of Agriculture, Agricultural Research Service, Davis, CA, United States

**Keywords:** uORF, amino acid metabolism, sugar metabolism, biotic and abiotic stress, plant growth and development

## Abstract

Sugar metabolism not only determines fruit sweetness and quality but also acts as signaling molecules to substantially connect with other primary metabolic processes and, therefore, modulates plant growth and development, fruit ripening, and stress response. The basic region/leucine zipper motif (bZIP) transcription factor family is ubiquitous in eukaryotes and plays a diverse array of biological functions in plants. Among the bZIP family members, the smallest bZIP subgroup, S1-bZIP, is a unique one, due to the conserved upstream open reading frames (uORFs) in the 5′ leader region of their mRNA. The translated small peptides from these uORFs are suggested to mediate Sucrose-Induced Repression of Translation (SIRT), an important mechanism to maintain sucrose homeostasis in plants. Here, we review recent research on the evolution, sequence features, and biological functions of this bZIP subgroup. S1-bZIPs play important roles in fruit quality, abiotic and biotic stress responses, plant growth and development, and other metabolite biosynthesis by acting as signaling hubs through dimerization with the subgroup C-bZIPs and other cofactors like SnRK1 to coordinate the expression of downstream genes. Direction for further research and genetic engineering of S1-bZIPs in plants is suggested for the improvement of quality and safety traits of fruit.

## Introduction

Plants have developed diverse mechanisms to regulate their biological and metabolic processes via transcription factor (TF) regulatory networks (Riechmann et al., 2000). Among the TF families, the basic leucine zipper (bZIP) family is present in all eukaryotes and is one of the largest and most diverse TF groups in higher plants. There are about four times more bZIP genes in the *Arabidopsis* genome than in the genomes of other model organisms such as *Saccharomyces cerevisiae*, *Caenorhabditis elegans*, and *Drosophila melanogaster* (Riechmann et al., 2000). Large numbers of bZIP TF family members have been found in many plant species including rice (Nijhawan et al., 2008), maize (Wei et al., 2012), tomato (Li D. et al., 2015), common wheat (Li X. et al., 2015), sorghum (Wang et al., 2011), soybean (Liao et al., 2008), banana (Hu et al., 2016a), cassava (Hu et al., 2016b), grape (Liu J. et al., 2014), peach (Wang et al., 2015), strawberry (Wang et al., 2015; Zhang et al., 2022), apple (Wang et al., 2015; Li et al., 2016), rapeseed (Zhou et al., 2017), radish (Fan et al., 2019), cucumber (Baloglu et al., 2014), tea plant (Xue et al., 2018), sweet potato (Yang Y. et al., 2019), watermelon/melon (Unel et al., 2019), Chinese jujube (Zhang et al., 2020a), pepper (Gai et al., 2020), Chinese pear (Manzoor et al., 2021), poplar (Zhao et al., 2021), quinoa (Li et al., 2020) and plum (Li et al., 2021).

The bZIP family is phylogenetically categorized into different groups, with different species having various members of homologs. For example, the *Arabidopsis* AtbZIP family members were systematically classified into 10 groups (A–I and S) based on conserved motifs (Jakoby et al., 2002). Subsequently, a more complete classification was expanded into 13 groups, designated as A-J, M, and S (Corrêa et al., 2008). The tomato SlbZIPs were classified as nine clades (Li D. et al., 2015). The cucumber CsbZIPs and sorghum SbbZIPs were separately categorized into six and seven groups (Wang et al., 2011; Baloglu et al., 2014). The bZIP family in both rice and maize has 11 groups which are the same as castor bean (Nijhawan et al., 2008; Wei et al., 2012; Jin et al., 2014). The plum PmbZIP proteins were divided into 12 groups (Li et al., 2021). Chinese pear PbbZIPs were categorized into 13 groups (Manzoor et al., 2021). Several interspecies clustering studies indicate that the S group found in *Arabidopsis* has especially high homology across different species (Li D. et al., 2015; Li et al., 2020; Manzoor et al., 2021), although some clades might be specific to *Arabidopsis* compared to peach, strawberry, and apple (Wang et al., 2015).

These classifications, phylogeny, and homology analyses define the possible biological roles of bZIPs in green plant evolution (Corrêa et al., 2008). Basic leucine zipper TFs orchestrate a diverse array of functions in multiple biological processes including flower development (Chuang et al., 1999; Walsh and Freeling, 1999; Strathmann et al., 2001; Abe et al., 2005; Wigge et al., 2005; Muszynski et al., 2006; Romera-Branchat et al., 2020) and pollen development (Gibalová et al., 2009; Iven et al., 2010), seed maturation (Izawa et al., 1994; Toh et al., 2012; Zinsmeister et al., 2016; Jain et al., 2018), senescence (Smykowski et al., 2015), light signaling (Chen et al., 2013; Abbas et al., 2014; Xu, 2020), anthocyanin and chlorophyll biosynthesis (An et al., 2017; Wang et al., 2020), nutrient signaling (Dröge-Laser and Weiste, 2018; Pedrotti et al., 2018; Yang Z. et al., 2019), hormone signaling such as salicylic acid, ABA, ethylene, auxin, and cytokinin (Singh et al., 2002; Li et al., 2011; Weiste and Dröge-Laser, 2014; Zong et al., 2016; Xu et al., 2018; Lv et al., 2019; Srivastava et al., 2019), sugar signaling (Kang et al., 2010; Ma et al., 2011; Thalor et al., 2012; Sagor et al., 2016), and abiotic/biotic stress signaling (Tsugama et al., 2012, 2016; Alves et al., 2013; Zong et al., 2016; Sun et al., 2017; Li et al., 2019; Yang J. et al., 2019; Carianopol et al., 2020) in plants.

Group S is the largest bZIP subgroup in several species such as *Arabidopsis* (Jakoby et al., 2002) and safflower (Li et al., 2020) and comprises three to four even smaller subgroups. In this review, we focus on the well-studied S1-bZIP subgroup, whose members contain unique conserved upstream open reading frames (uORFs) in the 5′ region of their transcripts and play important regulatory roles in many metabolic processes relating to fruit quality and stress responses. Our review aims to provide perspectives for further surveying the biological function, exploring regulatory mechanisms, and genome engineering the S1-bZIPs to obtain desirable traits for quality improvement in horticultural plants.

## Classification and Structure of S1-bZIPs

**FIGURE 1:**
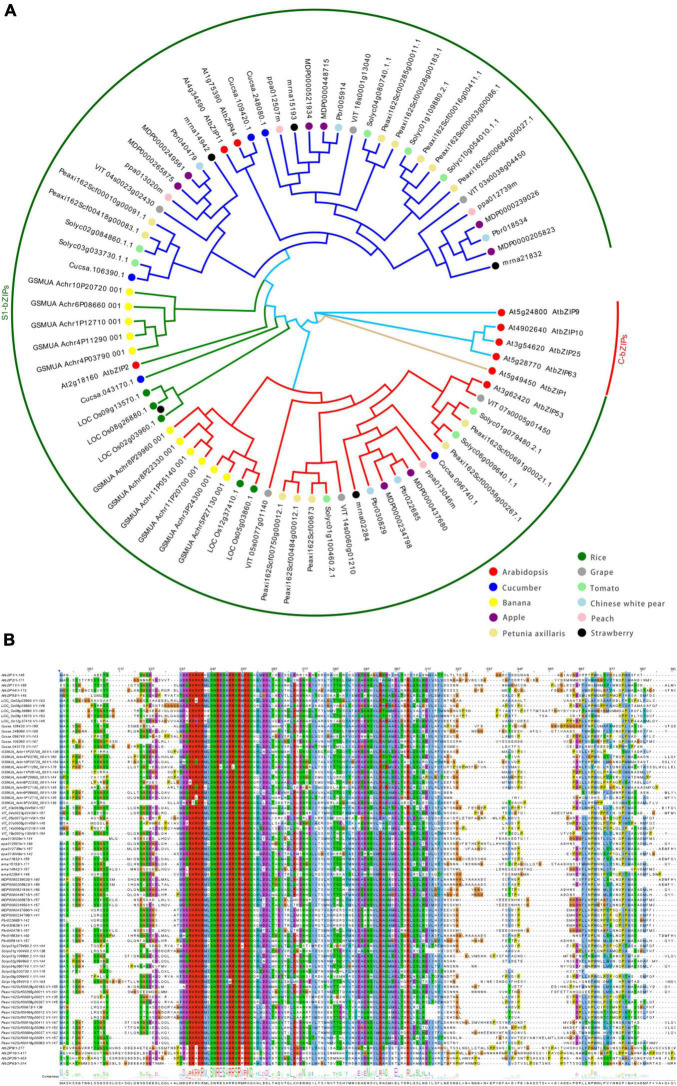
Phylogenetic analysis of S1-bZIPs in some species. **(A)** The phylogenetic tree was constructed by the neighbor-joining method (NJ) using MEGAx software. The phylogenetic trees were drawn with EvoView at the following URL: https://www.evolgenius.info/evolview/. Colored dots represent members from various species. The proteins were classified into six different clades. Each clade was assigned a different color according to their inclusion of each *Arabidopsis* S1-bZIP member. **(B)** The predicted amino acid sequences encoded by the *Arabidopsis* S1 and C group bZIP mORFs are aligned with the S1 homologs from other species using the multiple sequence alignment tools of ClustalW (Chenna et al., 2003) and the alignment results were displayed using Jalview (Waterhouse et al., 2009). The addition of the *Arabidopsis* C-bZIP serves as an outgroup.

Of the AtbZIPs, the 17 members of the S group are further separated into three subgroups based on homology: S1, S2, and S3 (Ehlert et al., 2006). The S1 subgroup (S1-bZIP) in *Arabidopsis* contains five members: AtbZIP1, −2, −11, −44, and −53. Recent studies indicate that other species, including many horticultural plants, also have multiple members of the S1-bZIP subgroup (Figure 1A and Supplementary Table 1). Like other bZIP members, those in the S1 subgroup are characterized by a conserved bZIP domain, composed of two functionally distinct motifs (a basic region and a leucine zipper) located on a contiguous α-helix. The basic region of −18 amino acids contains, sequentially, a nuclear localization signal and an invariant N-x7-R/K-x9 motif for DNA binding. This motif preferentially binds to the A-box, C-box, and G-box of target promoters which contain DNA sequences with an ACGT core (Jakoby et al., 2002; Dröge-Laser et al., 2018; Li et al., 2021). The leucine zipper comprises a heptad repeat of leucines or other numerous hydrophobic amino acids (L-x_6_-L-x_6_-L) (Figure 1B). Compared to other groups, members of the S group include the extraordinarily high number of eight hydrophobic amino acid repeats (Ehlert et al., 2006; Dröge-Laser et al., 2018) (Figure 1B). The two subunits form a zipper structure that binds DNA to form dimers through interactions with the hydrophobic sides of the helices (Jakoby et al., 2002). Of three S subgroups, only members of the S1 subgroup show specific heterodimerization with C group bZIP proteins (C-bZIPs), whereas weak homodimerization within members of the S1 subgroup is detected (Ehlert et al., 2006; Peviani et al., 2016). Phylogenetic analysis between S1 and C group bZIPs from angiosperms, gymnosperms, mosses, and algae suggests that the S1 and C groups evolved from a proto-S/C bZIP in algae species that homodimerized, which has since diverged into heterodimerizing pairs prior to the evolution of seeds plants (Peviani et al., 2016).

## Unique Upstream Open Reading Frame Structure and Translational Regulation Mechanism of S1-bZIPs

**FIGURE 2:**
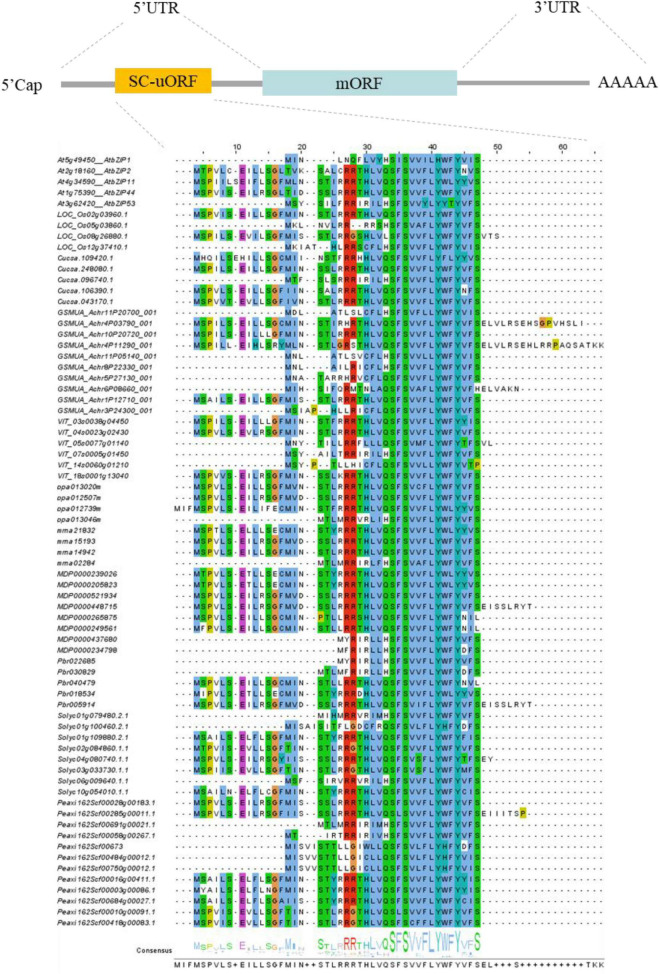
Full-length gene structure of the S1-bZIPs, and alignment of the highly conserved S1-bZIP uORFs encoding the sucrose control peptide (SC-peptide) from some species. Conserved amino acids are depicted in color.

Besides their common structural features, S1-bZIPs are unique in that they have an unusually long 5′-leader sequence in the upstream region of the main open reading frame (mORF) of the mRNA. This leader sequence contains several upstream open reading frames (uORFs) that encode small peptides (Dröge-Laser et al., 2018). Among those, the second uORF is conserved and encodes a Sucrose Control peptide (SC-peptide) of 28 residues, which regulates the translation of the mORF and reduces protein expression through a mechanism known as Sucrose-Induced Repression of Translation (SIRT), which contributes to sucrose homeostasis in the cells (Wiese et al., 2004; Rahmani et al., 2009). Here, we summarize uORFs of the S1-bZIP subgroup from different horticultural plants, including banana (Hu et al., 2016a), grape (Liu J. et al., 2014), apple (Wang et al., 2015), peach (Wang et al., 2015), cucumber (Baloglu et al., 2014), strawberry (Baloglu et al., 2014; Zhang et al., 2022), petunia (Sun et al., 2017), and white pear (Wu et al., 2013) (Figure 2 and Supplementary Table 1).

**FIGURE 3:**
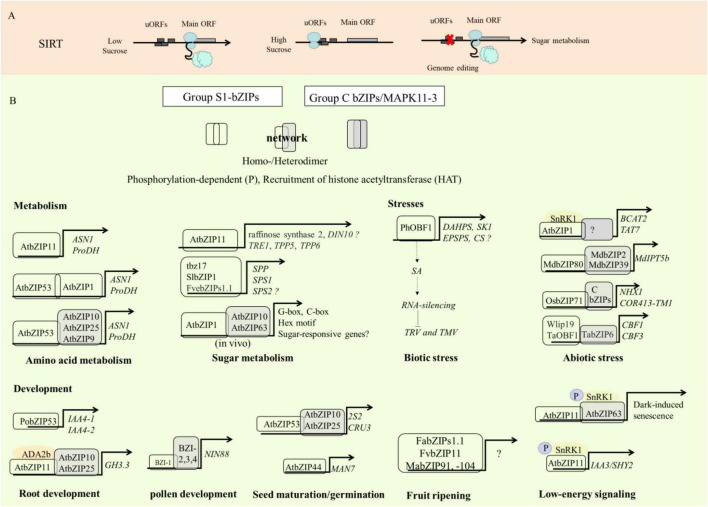
Multiple levels of regulation and biological function of S1-bZIPs. **(A)** Translation regulation of S1-bZIPs by SIRT. SIRT: Sucrose-induced repression of translation. **(B)** Biological function and target genes regulated by S1-bZIP. The regulated target genes by S1-bZIP (white) or heterodimers of S1-bZIPs and C-bZIPs (gray). Gene names: *ASN1*, *ASPARAGINE SYNTHETASE 1*; *ProDH*, *Proline Dehydrogenase*; *TRE1*, *Trehalase 1*; *TPP5/-6*, *trehalose-phosphate phosphatase 5*/-*6*; *SPP*, *sucrose-phosphatase*; *SPS1/-2*, *sucrose-phosphate synthase 1/-2*; *HXK1*, *hexokinase 1*; *DAHPS*, *3-Deoxy-D-arabino-heptulosonate 7-phosphate synthase*; *SK1*, *shikimate kinase 1*; *EPSPS*, *5-enolpyruvylshikimate 3-phosphate synthase*; *CS*, *chorismate synthase*; *BCAT2, BRANCHED-CHAIN AMINO ACID TRANSAMINASE2; TAT7*, *TYROSINE AMINOTRANSFERA- SE7; IPT5b*, *Isopentenyltransferase 5b*; *NHX1*, *Na+/H+exchanger 1*; *COR413-TM1*, cold acclimation protein; *CBF1*/-*3*, *C-repeat/DRE binding factor 1*/-*3*; ADA2b: transcriptional adapter ADA2b; *IAA4-1/4-2/-3*, *INDOL-3-ACETIC ACID INDUCIBLE 4-1/4-2/-3*; *SHY2*, *SHORT HYPOCOTYL 2*; *GH3.3*, *Indole-3-acetic acid-amido synthetase*; *NIN88*, *Defective invertase*; *2S2*, *SEED STORAGE ALBUMIN*; *CRU3*, *CRUCIFERIN* 3; *MAN7*, *endo-beta-mannase 7*; P, phosphorylation.

The regulation of gene expression involves different layers, including transcriptional and translational controls (van der Horst et al., 2020). Compared with transcriptional regulation, translational control allows more immediate responses to adjust protein expression and reprogram metabolism upon cellular signals or environmental stimuli (Jorgensen and Dorantes-Acosta, 2012; Chen et al., 2020). The translation process of mRNA includes four major steps: initiation, elongation, termination, and ribosome re-initiation (van der Horst et al., 2020). Translation initiation is the major step that determines the rate of protein biosynthesis and is regulated by multiple mechanisms (Sonenberg and Hinnebusch, 2009; Jackson et al., 2010; Hinnebusch et al., 2016; Zhang et al., 2019; van der Horst et al., 2020). uORFs have been suggested to play a critical role in regulating the translation of the mORF (Morris and Geballe, 2000; Kochetov, 2008; Ruiz-Orera and Albà, 2019). uORFs of S1-bZIPs are involved in the translational regulation in a SIRT manner (Jorgensen and Dorantes-Acosta, 2012; von Arnim et al., 2014). The SC-peptide encoded by the uORF in the 5′leader region of *AtbZIP11* is capable of repressing translation of the subsequent mORF in the presence of sucrose (Rahmani et al., 2009). High sucrose levels enhance ribosome stalling on the uORF, which results in poor translation of the mORF (Rook et al., 1998; Hummel et al., 2009; Peviani et al., 2016; Merchante et al., 2017; van der Horst et al., 2020) (Figure 3A). The members of the *Arabidopsis* S1-bZIP subfamily show similar responses to sucrose. Translation of *AtbZIP1*, *AtbZIP2*, *AtbZIP11*, *AtbZIP44* and *AtbZIP53* is downregulated by sucrose (Rook et al., 1998; Price et al., 2004; Kang et al., 2010). Transgenic seedlings with 35S:bZIP11 5′ leader::LUC show significantly reduced luciferase activities when treated with sucrose while those incubated in media lacking sucrose show two- to three-fold higher luciferase activities (Rahmani et al., 2009). SIRT-mediating S1-bZIP orthologs exist in all seed plants (Peviani et al., 2016). Previous research showed that amino acids such as serine, leucine, and tyrosine in the conserved peptide of uORF are essential for SIRT (Rahmani et al., 2009). However, it has been shown that expressing the gymnosperm 5′uORF sequence, which only contains the conserved leucine and tyrosine in *Arabidopsis* cells efficiently mediates the translational repression of the LUC reporter gene in response to sucrose (Peviani et al., 2016). This study suggests that the SIRT mechanism most likely depends on structural conformation, but not on recognition of specific sequence motifs (Peviani et al., 2016). Recently, interesting research conducted using gene-editing technology in strawberry demonstrated that uORFs are involved in regulating protein translation efficiency and sucrose content (Xing et al., 2020) (Figure 3A). In the study, to manipulate the SC-uORF of *FvebZIPs1.1*, the start codons of the uORF and the codons encoding a conserved pair of amino acid arginine within the SC-peptide were edited using the CRISPR/Cas9 system. Mutations in the start codons and the conserved C-terminal region of the SC-peptide significantly reduced translation of the SC-uORF. This consequently enhanced the translation efficiency of the downstream mORF. Seven novel alleles with C-to-T substitutions and small deletions within the uORF were identified. To test if phenotypic effects were additive in heterozygous and biallelic plants, 4000 T1 seedlings were generated by crossing the biallelic and homozygous T0 mutants to each other and to wild type. 35 novel genotypes were obtained in T1 and inherited in T2 generation. In comparison with wild-type fruits, the mutants had significantly higher levels of fructose, glucose, and total sugar contents, demonstrating that engineering the conserved SC-uORF of *FvebZIPs1.1* can increase the sugar content in strawberry (Xing et al., 2020). In addition, the citric acid content was slightly lower in the homozygous mutants than that in wild type. A continuum of gradual increase of sugar contents was generated in T1 by combining heterozygous, homozygous, and biallelic mutants, and inherited in T2 generation by propagating stolons of these T1 mutants, therefore confirming the transmissibility of novel genotypes and phenotypes from T1 to T2 by asexual propagation (Xing et al., 2020). Given that sugars can modulate multiple growth and development processes, the agricultural traits including leaf shapes, leaf areas, plant height, growth rates, pollination, fruit size and fruit weight were further evaluated in *FvebZIPs1.1* uORF mutants. Remarkably, editing SC-uORF does not severely impair plant growth. The agricultural traits in *FvebZIPs1.1* uORF mutants were similar to wild-type (Xing et al., 2020), whereas impaired phenotypes and retarded growth are observed in transgenic lines with the overexpression of *AtbZIP11*, *tbz17*, and *FvbZIP11* mORF (Ma et al., 2011; Thalor et al., 2012; Zhang et al., 2022). Taken together, this suggests a broad application of editing uORFs of S1-bZIPs for quality improvement in horticultural plants.

## S1-bZIPs Affect Amino Acid Metabolism

Amino acids are not only involved in plant response to stress but also influence fruit flavor (Keutgen and Pawelzik, 2008). For example, asparagine is present in almost all fruits and determines fruit flavor and quality in a concentration-dependent manner (Aisala et al., 2020). Glutamate is responsible for “umami” or savory taste (Lindemann, 2001). Glycine, alanine, serine, threonine, proline, glutamine, and lysine are highly correlated with sweetness (Sagor et al., 2016), while phenylalanine and tyrosine are bitter (Belitz et al., 2001). The molecular taste receptor, found in humans and rodents, responds to asparagine and aspartic acid (Nelson et al., 2002). Asparagine is considered to serve as a nitrogen storage molecule and synthesized at night under low-carbon conditions (Lam et al., 1994; Hanson et al., 2008). Asparagine and glutamate are synthesized from aspartate and glutamine through ASPARAGINE SYNTHETASE1 (ASN) (Lam et al., 1994; Hanson et al., 2008). A high sugar content represses the expression of *ASN* and reduces asparagine content (Lam et al., 1994). Likewise, proline levels change in response to energy levels. PROLINE DEHYDROGENASE (ProDH) converts proline to glutamate (Hayashi et al., 2000). Recent studies demonstrate that S1-bZIPs directly regulate the expression of *ProDH* and *ASN1 via* binding to the C-boxes, ACT motifs (ACTCAT), and G-boxes in their promoters, thereby influencing amino acid metabolism (Weltmeier et al., 2006; Hanson et al., 2008; Dietrich et al., 2011). Overexpression of *tbz17* mORF in tobacco significantly induces the expression of *ASN*, whereas silencing of *tbz17* represses the expression of *ProDH* and *ASN* (Thalor et al., 2012). One of the target genes of AtbZIP53 is *ProDH2* (Satoh et al., 2004). Overexpression of *SlbZIP1* and *AtbZIP11* mORFs in the transgenic tomato and *Arabidopsis* significantly up-regulates the expression of *ASN1* and *ProDH2* and affects amino acid contents (Hanson et al., 2008; Dietrich et al., 2011; Thalor et al., 2012; Sagor et al., 2016). For example, overexpression of *SlbZIP1* increases the content of alanine, aspartic acid, glutamate, serine, threonine, tyrosine, and total amino acid content. Energy deprivation induces the expression of *ASN1* and *ProDH*, which contributes to the recycling of amino acids to mitigate deficits of carbon, nitrogen, and energy (Dietrich et al., 2011). Many amino acid catabolism related genes induced by *AtbZIP11* are largely repressed by treatments with sugar (Hanson et al., 2008). Moreover, under high-sucrose conditions, the translation of AtbZIP11 is inhibited *via* a uORF (Hanson et al., 2008; Yamashita et al., 2017). These findings indicate that *ASN1* and *ProDH* are ultimately regulated in a sugar-dependent manner, with AtbZIP11 acting as the link between sugar signaling and amino acid/nitrogen metabolism (Hanson et al., 2008). Additionally, AtbZIP1 and AtbZIP53 are also involved in modulating amino acid metabolism during stress responses (Baena-González et al., 2007; Hartmann et al., 2015). In *Arabidopsis*, it has been demonstrated that AtbZIP53 preferentially forms heterodimers with group C-bZIP members like AtbZIP9, AtbZIP10, and AtbZIP25 for controlling the gene expression of *ASN1* and *ProDH* (Weltmeier et al., 2006; Garg et al., 2019) (Figure 3B). However, the interacting partners between the S1- and C-bZIPs are not identified in many other crops and need to be investigated in the future.

## S1-bZIPs Affect Sugar Metabolism

Overexpression of S1-bZIP mORFs induces sugar-related gene expression and increases sugar content (Figure 3B). Previous studies have shown that overexpression of *tbz17* and *SlbZIP1* mORF up-regulates the expression level of genes encoding sucrose phosphate synthase (*SPS*) and sucrose phosphate phosphatase (*SPP*), whereas silencing *tbz17* down-regulates the expression of these genes (Thalor et al., 2012). Knockout and overexpression of *AtbZIP1* affects sugar-responsive gene expression (Kang et al., 2010). Furthermore, it has been demonstrated that constitutive expression of the *S1-bZIP1*s such as *tbz17 and AtbZIP11* mORF significantly increases the sucrose concentration in transgenic lines (Ma et al., 2011; Thalor et al., 2012). Interestingly, the contents of glucose and fructose were significantly increased and the citric acid content was significantly decreased in transgenic plants with overexpression *AtbZIP11* (Ma et al., 2011). The induction of the *AtbZIP11* mORF also results in the up-regulation of genes associated with the metabolism of trehalose, myo-inositol and raffinose. Transgenic *Arabidopsis* lines overexpressing *AtbZIP11* showed decreased contents of the trehalose-6-phosphate (T6P), limiting the plant’s ability to use available sugars, thereby slowing plant growth. This growth inhibition in *Arabidopsis* cannot be reversed by the exogenous application of metabolizable sugars such as glucose and sucrose (Ma et al., 2011). The use of the fruit-specific E8 promoter to drive overexpression of *SlbZIP1* increases the sugar contents in tomato while avoiding growth impairment (Sagor et al., 2016). Remarkably, sucrose contents were approximately sixfold higher in transgenic lines with approximately 1.5-fold higher fructose, glucose, and total sugar contents than in wild type. Similar effects such as significantly increased glucose and fructose contents and significantly reduced citric acid content were observed in mutants with enhanced *FvebZIP1.1* mORF protein expression due to the uORFs mutation (Xing et al., 2020). In a recent study, heterologous overexpression of strawberry *FvbZIP11* affects fruit quality and flavor in tomato (Zhang et al., 2022). In comparison with wild type, the total soluble solid was significantly increased at the breaker, pink and red ripe stages in three transgenic tomato lines. The soluble sugar (SS) content was significantly accumulated at 30–50 days after anthesis in transgenic line 6. In addition, the titratable acid content (TTA) was significantly reduced at 30 days after anthesis, while SS/TTA ratio was significantly increased from 20 to 50 days after anthesis in the transgenic tomato line (Zhang et al., 2022). Taken together, these studies demonstrate that the S1-bZIPs play important roles in the regulation of sugar metabolism for quality improvement in plants.

## Regulatory Roles of S1-bZIPs in Response to Biotic and Abiotic Stresses

S1-bZIPs play an essential role in plant adaptation to unfavorable conditions (Alves et al., 2013; Sornaraj et al., 2016; Noman et al., 2017) (Figure 3B). It has been documented that S1-bZIPs play important roles in plant innate immunity, especially against attack by various pathogens (Lee et al., 2002; Alves et al., 2013), and in response to abiotic stresses, such as cold (Shimizu et al., 2005; Kobayashi et al., 2008), drought (Ditzer and Bartels, 2006; Shekhawat and Ganapathi, 2014), and salinity (Hartmann et al., 2015). It has been demonstrated that the C-/S1-bZIP-SnRK1 complex participates in the reprogramming of primary metabolism related to carbohydrate and amino acid and induces salt stress tolerance through ABA-independent signaling in *Arabidopsis* roots (Hartmann et al., 2015). Similarly, C-/S1-bZIP-SnRK1 signaling is involved in defenses against biotic stresses, which are also energy-consuming processes that require metabolic readjustment in plants (Hulsmans et al., 2016). Research in our laboratory has suggested that petunia PhOBF1, a homolog of AtbZIP11, is involved in plant defenses against a wide range of viral pathogens (Sun et al., 2017). In the study, silencing *PhOBF1* resulted in the reduction of RNA silencing-related gene expression, including RNA-dependent RNA polymerases, Dicer-like RNase III enzymes, and Argonaut. *PhOBF1*-RNAi plants displayed a compromised resistance to tobacco rattle virus (TRV) and tobacco mosaic virus (TMV). On the other hand, overexpression of *PhOBF1* in petunia enhances resistance to these virus infections. Interestingly, *PhOBF1*-silenced petunia lines produced much lower levels of the compounds associated with the shikimate and phenylpropanoid pathways such as free salicylic acid (SA), salicylic acid glucoside, and phenylalanine, but much higher levels of those were detected in *PhOBF1* overexpressing plants (Sun et al., 2017). Intriguingly, *PhbZIP44*, a paralog of *PhOBF1* appears to be unable to participate in this antiviral process, suggesting functional diversity and specificity among the S1-bZIPs (Sun et al., 2017).

In *Arabidopsis*, S1-bZIPs AtbZIP11/ATB2, AtbZIP44, AtbZIP2/GBF5, and AtbZIP53 can bind to a 6-bp *cis*-acting element (ACTCAT) located in the promoter of *ProDH* (Satoh et al., 2004), which is responsive to hypoosmolarity and proline. AtbZIP53 directly and strongly promotes hypoosmolarity-induced transcription of *ProDH*, which is enhanced by the synergistic interplay between AtbZIP53 and the group C member AtbZIP10 (Satoh et al., 2004; Weltmeier et al., 2006). Analysis of transcriptome data has revealed the complexity of the response to abiotic stresses by S1-AtbZIPs. For instance, the transcript level of *AtbZIP53* was found to be strongly up-regulated by salt stress in roots and by osmotic stress in green tissues. Cold, osmotic, and salt elicitors were found to remarkably increase the expression of *AtbZIP1* in roots and *AtbZIP11* in green tissues but inhibit the expression of *AtbZIP2* in green tissues. *AtbZIP44* shows a solid and specific response to cold stress in the root and to salinity in green tissues (Kilian et al., 2007; Weltmeier et al., 2009). The expression of *AtbZIP1* in roots was significantly induced by salt treatment. *Arabidopsis bzip1 bzip53* double mutant reprograms carbohydrate and amino acid metabolism to help roots adapt to salt stress. Furthermore, AtbZIP1 binds the promoter of *BCAT2* and *TAT7* and plays a role as a signalling module of SnRK1-bZIP1 under salt stress. This pathway is independent of ABA-SnRK2-AREB signaling pathways, whereas *bZIP53* transcription partially depends on the SnRK2/AREB pathway (Hartmann et al., 2015). In tomato, *SlbZIP1* increases salt tolerance by increasing the gene expression related to ABA biosynthesis and signal transduction (Zhu et al., 2018). In response to water deficiency, two cucumber S1-bZIP member (*CsbZIP6* and *CsbZIP30*) transcripts accumulated in the root but decreased in leaves (Baloglu et al., 2014). Likewise, in sweet potato, the expression of *IbbZIP1* is highly induced by treatments with NaCl and ABA. Abiotic stress-related genes are significantly up-regulated in the transgenic *Arabidopsis* overexpressing *IbbZIP1*, suggesting the role of *IbbZIP1* in salt and drought tolerance (Kang et al., 2019). In apple, an S1-bZIP, *MdbZIP80*, has been shown to negatively regulate cytokinin-mediated drought and salt tolerance (Feng et al., 2021). This study shows that MdbZIP80 specifically heterodimerizes with C-bZIPs MdbZIP2 and MdbZIP39. The formed C-/S1-bZIP complex then binds to the ACTCAT motif in the promoter of *MdIPT5b*, a gene encoding the rate-limiting enzyme isopentenyltransferase in the cytokinin biosynthesis pathway, thereby suppressing its expression. This leads to drought and salt stress response through the cytokinin pathway by delaying drought-induced premature leaf senescence by reducing oxidative damage and maintaining plant growth (Feng et al., 2021). Another study demonstrates that low temperature stress induces *mlipl5* expression, and the protein subsequently binds to the promoter region of *Adh1* (Kusano et al., 1995). Interestingly, mechanical damage in tea leaves leads to the activation of S1-bZIPs such as *CsbZIP2*, *−11*, *−14*, *−16*, *−20*, *−21*, *−28* and *−30* (Xue et al., 2018). Overall, it appears that the expression levels of these *S1-bZIPs* respond to stress signals in a tissue-specific manner. The members of S1-bZIP share partially redundant functions but play a role in unique regulatory mechanisms. Generally, the S1- and C-AtbZIPs heterodimerize to mediate stress signal transduction cascades. For example, S1-bZIP AtbZIP53 forms heterodimers with group C-bZIP members such as AtbZIP10 or AtbZIP25 and increases DNA binding activity, resulting in strong activation of the target genes. These heterodimers can also form tertiary complexes with the non-bZIP protein ABI3 (ABSCISIC ACID INSENSITIVE 3) to play a synergistic role in target gene expression (Alonso et al., 2009; Weltmeier et al., 2009); however, it needs to be demonstrated whether other members of S1-bZIP such as AtbZIP1 heterodimers are formed under stress conditions (Hartmann et al., 2015).

The S1-bZIP gene *low-temperature-induced protein 19* (*lip19*) is significantly induced by low temperature in monocots (Shimizu et al., 2005; Kobayashi et al., 2008; Cai et al., 2018). The LIP19 protein appears to be unable to form homodimers and bind to DNA in rice (Kobayashi et al., 2008). However, the counterpart of LIP19 proteins in maize and wheat can form homodimers and bind to *cis*-elements in DNA sequences (Kobayashi et al., 2008; Cai et al., 2018). The WLIP19 can heterodimerize with wheat TaOBF1, another low temperature-responsive S1-bZIP member. The stable heterodimerization between LIP19-type and OBF1-type proteins seems to induce the expression of target genes in response to different abiotic stresses, especially cold stress (Shimizu et al., 2005; Kobayashi et al., 2008; Cai et al., 2018). However, there is no definitive evidence showing that the formation of heterodimers or homodimers between WLIP19 and TaOBF1 directly affects the expression of the downstream stress-responsive genes including *COR* (cold-responsive) and *LEA* (late embryogenesis-abundant) genes (Lee et al., 2002). Recent research indicates that a group C-bZIP TabZIP6 dimerizes with WLIP19, TaOBF1, or itself and then binds to the promoters of genes encoding CBFs (C-repeat binding factors), resulting in inhibition of their expression. These dimers can also inhibit the expression of some *COR* genes (Liu C. et al., 2014). Rice S1-bZIP plays a vital role in ABA-mediated drought and salt stress response. One of the S1-bZIPs, OsbZIP71, appears to be able to form homodimers and heterodimers with group C-bZIP members OsbZIP15, OsbZIP20, OsbZIP33, and OsbZIP88. It has been speculated that these heterodimers help OsbZIP71 bind to the promoters of its target genes, *OsNHX1*, and *COR413-TM1* because OsbZIP71 on its own has weak DNA-binding activity to the G-box element and no transcriptional activation activity (Liu C. et al., 2014). Thus, the interplay between C-group and S1-subgroup is proposed to affect plant response to stress.

## Biological Roles of S1-bZIPs as Regulators of Plant Growth and Development

Plant growth and development are tightly interlinked with the control of metabolism, especially energy homeostasis. Transient energy deprivation causes plants to adjust their metabolism to adapt to daily light/dark cycles and unpredictable environmental changes. It has been proposed that the Snf1-related kinase 1 (SnRK1) and Target of Rapamycin (TOR) kinase function to reprogram plant metabolism in response to the energy status (Baena-González et al., 2007; Hulsmans et al., 2016). Evidence suggests that SnRK1 mediates the phosphorylation of S1-bZIPs to control plant growth and development under starvation and nutrient-replete conditions (Lastdrager et al., 2014) (Figure 3B). As the transcriptional regulators downstream of SnRK1, AtbZIP11 can directly control a subset of SnRK1-dependent genes *via* binding to G-box elements in their promoter regions (Pedrotti et al., 2018). Furthermore, heterodimerization between group C- and S1-bZIPs is enhanced by the phosphorylation of group C-bZIPs by SnRK1. Phosphorylation of AtbZIP63 provides the structural basis for forming the AtbZIP63-AtbZIP1-SnRK1/AtbZIP63-AtbZIP11-SnRK1 complex and ultimately leads to the adjustment of metabolism to ensure plant survival under low energy conditions (Pedrotti et al., 2018). Notably, the formation of the complex is dependent on the SnRK1-specific phosphorylation sites, which are pivotal for the function of AtbZIP1 and AtbZIP53 (Hartmann et al., 2015). Additionally, the identification of many SnRK1-independent genes regulated by AtbZIP11 indicates a function of AtbZIP11 beyond SnRK1 signaling (Dröge-Laser et al., 2018). It seems that heterodimers within the C-/S1-bZIP network function as a hub to integrate SnRK1-dependent and -independent signals to adjust growth/development and stress responses (Mair et al., 2015). Recent studies showed that S1-bZIPs regulate the root apical meristem size through controlling polar auxin flux (Weiste et al., 2017). Under low-energy conditions, *AtbZIP2*, *AtbZIP11*, and *AtbZIP44* directly activate the transcription of *INDOLE-3-ACETIC ACID PROTEIN 3/SHORT HYPOCOTYL 2* (*IAA3*/*SHY2*), a negative regulator of auxin signaling, which leads to the down-regulation of *PIN-FORMED (PIN)* genes, limiting polar auxin transport to the root tip and blocking auxin-driven primary root growth (Weiste et al., 2017).

S1-bZIPs play essential roles in plant growth and development, especially seed maturation, root growth, and flower development (Figure 3B). For example, the transcript abundance of *AtbZIP53* is markedly induced during the late stages of seed development (Weltmeier et al., 2009). AtbZIP53 enhances the gene expression associated with seed maturation by specific heterodimerization with group C-bZIPs (Alonso et al., 2009). AtbZIP11 and AtbZIP44 play a role in embryogenesis. *AtbZIP44* shows high transcript levels at the early stage of seed development and is involved in micropylar endosperm loosening and seed coat rupture via its interaction with the promoter of *AtMAN7* (Weltmeier et al., 2009). The *atbzip44* knock-out mutant shows slower germination and reduced expression of *AtMAN7* (Iglesias-Fernández et al., 2013). In *Populus*, the binding of poplar bZIP53 to the promoter of *IAA4-1* and *IAA4-2* inhibits adventitious root development (Zhang et al., 2020b). In horticultural plants, three S1-bZIP members (*VvbZIP07*, *14*, and *47*) are highly expressed in grape seed (Liu J. et al., 2014), but their regulatory mechanisms have yet to be elucidated. Other studies have shown that S1-bZIPs are related to floral development. For example, *CsbZIP-06* is highly expressed in female cucumber flowers and ovaries (Baloglu et al., 2014). Transgenic lines overexpressing mORF of *BZI-4* show reduced flower size and impaired pollen development (Iven et al., 2010). Overexpressing *AtbZIP1*, *AtbZIP53*, *tbz17*, *MusabZIP53*, and *FvbZIP11* shortened internode length, and stunted vegetative growth (Alonso et al., 2009; Dietrich et al., 2011; Thalor et al., 2012; Shekhawat and Ganapathi, 2014). *FabZIPs1.1* and *FvbZIP11* have been shown to be involved in fruit ripening in strawberry (Chen et al., 2020; Zhang et al., 2022). Banana *MabZIP91* and *MabZIP104*, which belong to S1-bZIP subgroup, showed high transcript abundance during fruit development and ripening (Chen et al., 2020). These studies illustrate the various roles of S1-bZIPs as a regulator of plant growth and development (Figure 3B).

## Concluding Remarks and Future Perspectives

The S1-bZIP subgroup, with their functional diversity in all plants, reflects their importance as regulators. The literature covered in this review suggests that the small but unique and crucial S1-bZIP transcription factors play essential roles in the balance of carbon and amino acid metabolism, plant growth and development, and stress responses (Figure 3). S1-bZIPs also play important roles in regulating fruit quality and stress response. Through heterodimerization with group C-bZIPs, S1-bZIPs orchestrate an array of downstream transcriptional and metabolic control. However the C group bZIP dimerization partners of many S1-bZIPs have yet to be identified. The S1-bZIPs regulate sugar signaling and amino acid metabolism under energy-deprived conditions, which involves the Sucrose Induced Repression of Translation mechanism of the uORFs and through interaction with the SnRK1 pathway. However, further research is needed to explore whether and how SnRK1 and TOR kinase interact with C- and S1-bZIPs complex. The SC-uORF negatively regulates the translation of S1-bZIP mORFs and, in turn, downstream targets of the S1-bZIPs, which further affect fruit quality and other metabolite biosynthesis. Evidence suggests that overexpression of S1-bZIP mORFs significantly increased the fruit sugar content and sweetness, showing the potential for improvement of fruit quality (Thalor et al., 2012; Sagor et al., 2016; Chen et al., 2020; Zhang et al., 2022). In addition, functional diversity and specificity among the S1-bZIPs need to be further defined. Using substitution of conserved amino acid residues in the DNA-binding domain could be a useful approach to clarify specific interconnections among S1-bZIPs and their dimerization partners in horticultural plants (Garg et al., 2019). Using CRISPR technology to create indel mutations in uORF start codons or enhancing the expression of S1-bZIPs using fruit-specific promoters could provide broad applications to control the levels of sucrose and other nutrients for the improvement of the quality of fruits, vegetables, and flowers, and to improve stress response without the detrimental effects on plant growth and development in horticultural plants (Corrêa et al., 2008; Shipman et al., 2021).

## Author Contributions

HW, YZ, AN, and C-ZJ collected data and wrote and revised the manuscript. All authors contributed to the article and approved the submitted version.

## Conflict of Interest

The authors declare that the research was conducted in the absence of any commercial or financial relationships that could be construed as a potential conflict of interest.

## Publisher’s Note

All claims expressed in this article are solely those of the authors and do not necessarily represent those of their affiliated organizations, or those of the publisher, the editors and the reviewers. Any product that may be evaluated in this article, or claim that may be made by its manufacturer, is not guaranteed or endorsed by the publisher.
